# A prospective study of time to healing and hypertrophic scarring in paediatric burns: every day counts

**DOI:** 10.1186/s41038-016-0068-2

**Published:** 2017-01-19

**Authors:** Elizabeth Chipp, Lisa Charles, Clare Thomas, Kate Whiting, Naiem Moiemen, Yvonne Wilson

**Affiliations:** Burns Centre, Birmingham Children’s Hospital, Steelhouse Lane, Birmingham, UK

**Keywords:** Scar, Hypertrophic, Burn, Time to healing, Paediatric, Skin type

## Abstract

**Background:**

It is commonly accepted that burns taking longer than 3 weeks to heal have a much higher rate of hypertrophic scarring than those which heal more quickly. However, some of our patients develop hypertrophic scars despite healing within this 3-week period.

**Methods:**

We performed a prospective study of 383 paediatric burns treated non-operatively at a regional burns centre over a 2-year period from May 2011 to April 2013. Scar assessment was performed by a senior burns therapist using the Vancouver Scar Scale.

**Results:**

Overall rates of hypertrophic scarring were 17.2%. Time to healing was the strongest predictor of developing hypertrophic scarring, and the earliest hypertrophic scar developed in a patient who was healed after 8 days. The risk of hypertrophic scarring was multiplied by 1.138 for every additional day taken for the burn wound to heal. There was a trend towards higher rates of hypertrophic scarring in non-white skin types but this did not reach statistical significance.

**Conclusions:**

The risk of hypertrophic scarring increases with every day and, therefore, every effort should be made to get the wound healed as quickly as possible, even within the traditional 3-week period usually allowed for healing. We believe that the traditional dogma of aiming for healing within 3 weeks is overly simplistic and should be abandoned: in paediatric burns, every day counts.

**Trial registration:**

Not applicable.

## Background

Hypertrophic scarring (HTS) following burn injury is a common problem which adds significant morbidity to a group of patients who are already dealing with a potentially devastating and life changing injury. Keloid and hypertrophic scars are known to have a negative impact on quality of life [1] and may require further surgical or non-surgical intervention.

Previous studies have found an incidence of HTS of 30–72% following burn injury [2]. Despite this, there is surprisingly little literature regarding the incidence or potentially modifiable risk factors. This is compounded by the fact that there is neither a single agreed definition of HTS nor a single best method for assessing burn scars; the Patient and Observer Scar Assessment Scale (POSAS) and Vancouver Scar Scale (VSS) are the most frequently used of the many scar assessment scales available [3]. It is therefore difficult to compare studies in order to get an idea of the true impact of HTS in this complex and heterogeneous group of patients.

It is commonly accepted that burns taking longer than 3 weeks to heal have a much higher rate of hypertrophic scarring than those which heal more quickly. For this reason, it is usually recommended that burns not expected to heal within a 3-week period are treated with excision and grafting [4, 5].

However, we are aware that some of our patients appear to develop troublesome and symptomatic hypertrophic scars despite healing within this 3-week period. We hypothesised that the goal of wound healing within 3 weeks was overly simplistic and may not apply to all patients. We considered whether scarring after burn injury was also influenced by phenotypic skin type, anticipating higher rates of hypertrophic scarring in non-white skin types.

### Aims

The main aim of this study was to determine whether incidence of HTS varied according to both the time taken for the burn to heal and the skin type of the patient. A secondary aim was to establish the rate of hypertrophic scarring in paediatric patients treated non-operatively in our centre.

## Methods

We conducted a prospective longitudinal observational study of children treated within a regional paediatric burn centre. Inclusion criteria were patients aged less than 16 years, presenting with acute burn injuries, who were managed without surgical intervention. Patients treated surgically were excluded and will be considered in a separate study. Those who failed to complete their treatment and follow-up, or who were followed up elsewhere, were also excluded. The study was approved by the hospital research and development group, and parents were asked to give written consent for both data collection and photography. Data was collected prospectively over a 2-year period from May 2011 to April 2013.

Data collected for each patient included age, causation, size and site of the burn injury and Fitzpatrick skin type [6]. Each patient or parent was also asked about any first aid performed and history of previous hypertrophic scarring. Patients were followed prospectively, and progress of wound healing and any clinical signs of infection were assessed at each dressing change. Standard practice in our centre is to dress burn wounds with a silver-based dressing, except for superficial burns >5% total body surface area (TBSA) where Biobrane is applied. Day of healing was recorded as the first attendance for review when the wound had completely healed and there was no further necessity for dressings. As outpatients were not reviewed daily, the first appointment at which the wound had completely healed was recorded as the actual day of wound healing. Digital colour photographs were taken of each burn site at the time of initial assessment and at each dressing change until the wound had healed. An experienced burns therapist assessed scarring using the modified Vancouver Scar Scale (mVSS) [7, 8]. For the purposes of this study, a hypertrophic scar was defined as one which was raised by at least 2 mm and had a total mVSS of 5 points or more. Where a patient had more than one mVSS recorded during their follow-up, the highest value was used.

Statistical analysis was undertaken by an independent statistician who was not involved in the clinical care of the patient. Analyses were performed using SPSS Statistics for Windows, Version 22.0 (Armonk, NY: IBM Corp). Continuous variables were summarised as means and ranges and categorical variables as counts and percentages. Binary logistic regression analysis was used to estimate the risk of HTS based on time to healing (as a continuous variable) and also to perform a multivariable analysis by including skin type in the model as well.

## Results

Data was collected from all patients who were treated non-surgically over a 2-year period from May 2011 to April 2013. Three hundred and eighty three patients had complete sets of data available for analysis. Patients were divided into groups according to time to healing (less than 8 days, 8–14 days, 15–21 days, greater than 21 days) and skin type (Fitzpatrick type 1–6).

There were 383 children; 248 male and 135 female patients. Mean age was 3.28 years (range 2 days to 15.6 years). Mean TBSA was 2.33% (range 0.25–40%), in keeping with conservatively managed burns that were treated largely on an outpatient basis. The majority of burns (91.4%) were caused by either scald or contact with flame burns accounting for 2.9% of injuries. The sites of the burn injuries were typical of this mostly pre-school age group with the upper limb and anterior trunk accounting for nearly two thirds of the total injuries seen (Table 1).

**Table 1:** Site of burn injury

Site of burn injury	Number (%)
Hand	94 (24.5)
Upper limb (excluding hand)	72 (18.8)
Anterior trunk	70 (18.3)
Lower limb (excluding foot)	55 (14.4)
Head and neck	46 (12.0)
Foot	32 (8.4)
Posterior trunk	9 (2.3)
Buttock/perineum	5 (1.3)

Thirty-three patients healed in less than 8 days, 171 healed in 8–14 days, 104 healed in 15–21 days and 75 healed after more than 21 days. The overall incidence of hypertrophic scarring in our patients was 17.2%; 66 of the 383 patients had a raised scar with a mVSS of at least 5 at some point during their follow-up. Healing took between 5 and 62 days, and the earliest hypertrophic scar was seen in a wound which healed after 8 days.

The results show a correlation between time to healing and the formation of hypertrophic scars: the rates were 0% in those patients who healed in less than 8 days, 6.4% for 8–14 days, 13.5% for 15–21 days and 56.0% in those who took longer than 21 days to heal. Overall, 8.1% of wounds which took less than 21 days to heal developed HTS, compared to 56% of wounds which took longer than 21 days to heal.

**Fig. 1:**
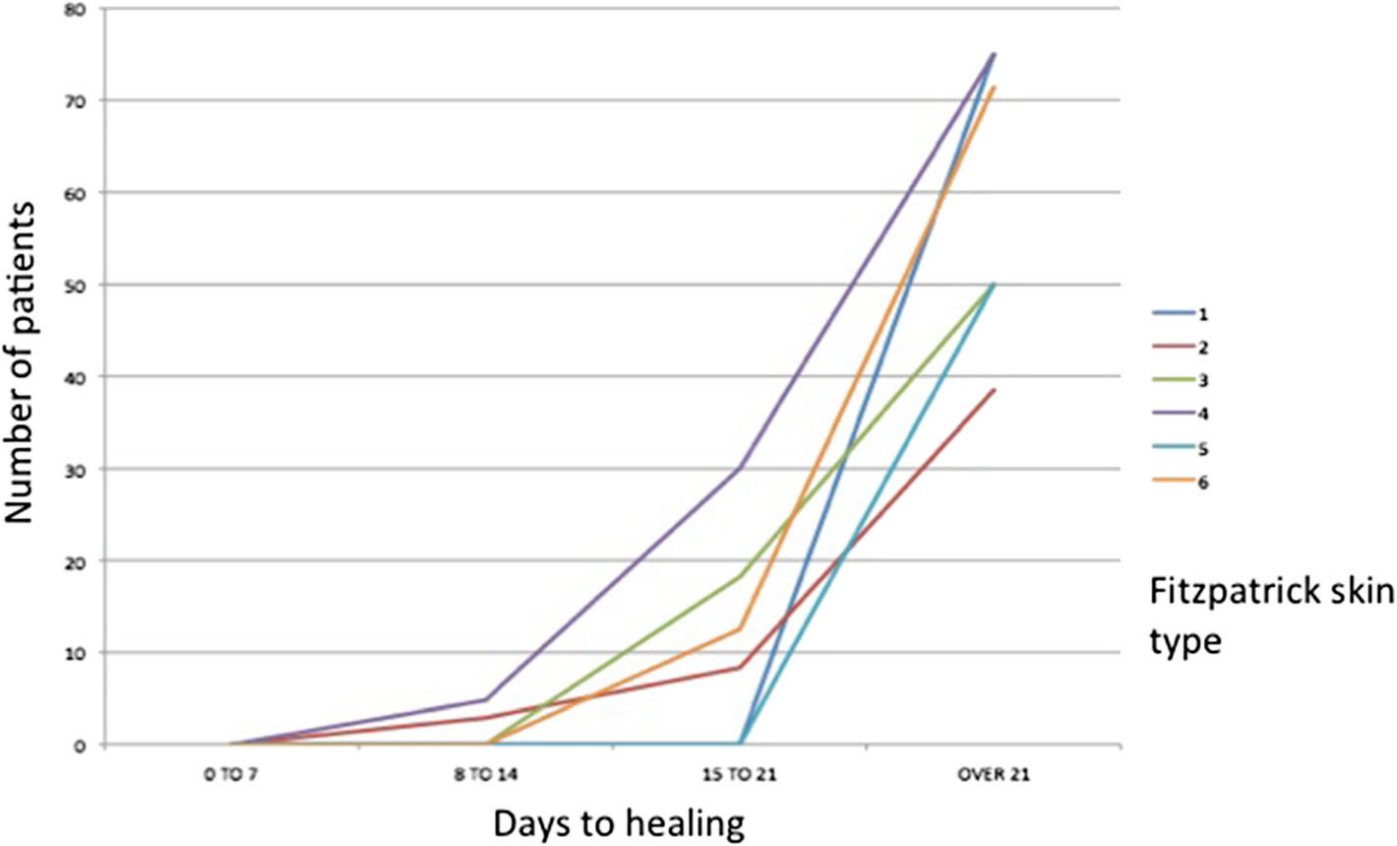
Rate of HTS by skin type and time to healing

The incidence of HTS varied by both time to healing and skin type. Patients with white skin (Fitzpatrick type 1–3) had rates of HTS of less than 15% when healed before 21 days, in keeping with previous studies [4, 5]. However, patients with Asian and black skin (Fitzpatrick type 4–6) showed higher rates of HTS even when the burn wound was healed before 21 days (Table 2). Patients with type 4 skin had the highest rates of HTS overall (24.1% incidence) and the highest rate at each time point up until 21 days. Rates of HTS in these patients were also higher in burns which healed before 21 days; 12.9% of patients with type 4 skin were compared to rates of 0–9.4% in the other groups. These trends are shown in Table 3 and Fig. 1. Although there appeared to be a trend towards higher rates of HTS in different skin types, this did not reach statistical significance (*P* = 0.184)

**Table 2:** Incidence of HTS by time to healing and skin type

Time to healing (days)	Type 1 (*n* = 40)	Type 2 (*n* = 104)	Type 3 (*n* = 61)	Type 4 (*n* = 106)	Type 5 (*n* = 36)	Type 6 (*n* = 36)	Overall (*n* = 383)
<8	0/4 (0%)	0/6 (0%)	0/5 (0%)	0/10 (0%)	0/6 (0%)	0/2 (0%)	0/33 (0%)
8–14	0/20 (0%)	4/53 (7.5%)	2/24 (8.3%)	4/46 (8.7%)	1/14 (7.1%)	0/14 (0%)	11/171 (6.4%)
15–21	1/10 (10.0%)	4/27 (14.8%)	0/19 (0%)	7/29 (24.1%)	2/12 (16.7%)	0/7 (0%)	14/104 (13.5%)
>21	2/6 (33.3%)	8/18 (44.4%)	7/13 (53.8%)	15/21 (71.4%)	3/4 (75.0%)	7/13 (53.8%)	42/75 (56.0%)

**Table 3:** Incidence of HTS by skin type and healing before/after 21 days

Time to healing (days)	Type 1 (*n* = 40)	Type 2 (*n* = 104)	Type 3 (*n* = 61)	Type 4 (*n* = 106)	Type 5 (*n* = 36)	Type 6 (*n* = 36)	Overall (*n* = 383)
<21	1/34 (2.9%)	8/86 (9.3%)	2/48 (4.2%)	11/85 (12.9%)	3/32 (9.4%)	0/23 (0%)	25/308 (8.1%)
>21	2/6 (33.3%)	8/18 (44.4%)	7/13 (53.8%)	15/21 (71.4%)	3/4 (75%)	7/13 (53.8%)	42/75 (56.0%)

Figure 1 shows that the incidence of HTS is highest in type 4 skin at all time points, although this difference did not reach statistical significance.

Further analysis was undertaken by an independent statistician. A binary logistic regression was performed with hypertrophic scarring (HTS) as the dependent variable. Time to healing did produce a statistically significant result, independent of skin type. The odds ratios for days to healing is 1.138, (95% CI 1.100–1.177, *P* < 0.001), i.e. the risk of developing HTS is multiplied by 1.138 for every additional day taken to heal.

**Fig. 2:**
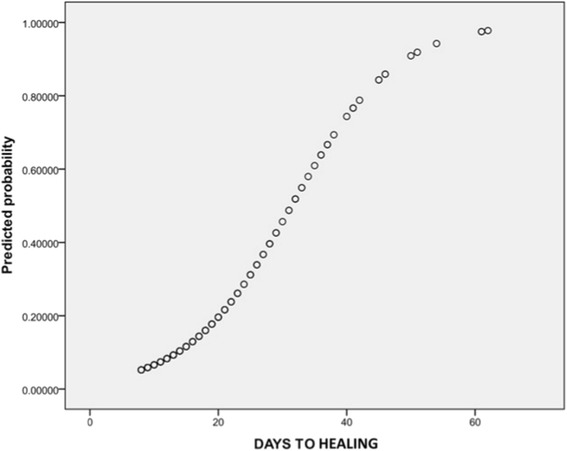
Predicted risk of HTS versus time to healing

The probability of developing HTS is depicted in graph form below (Fig. 2). The group who healed in less than 8 days and experienced no hypertrophic scarring are excluded from this graph. Each patient who healed after 8 days or more is plotted as a single point on the graph which illustrates the risk of developing HTS at any given time point.

## Discussion

Hypertrophic scars are an unwelcome and common sequelae, following even a minor burn. These scars cause significant morbidity in terms of discomfort, pain, itching, concerns about appearance and have been shown to have a negative effect on quality of life [1]. This is especially true for children where treatment such as intralesional steroid injections may require procedures under general anaesthetic, or repeated trips to hospital for scar therapy, causing disruption to family and school life.

Although generally accepted to be a red and raised scar which does not extend outside the boundaries of the original injury, the lack of a formal definition of hypertrophic scars makes it difficult to accurately determine their incidence. Given these limitations, Lawrence et al. reported an incidence of HTS of between 32 and 72% from their systematic review, although they found only seven studies which met their inclusion criteria [2]. Several published studies consist of retrospective case note reviews where any documentation of a scar which is red or raised constitutes a diagnosis of HTS [5, 9]. Other studies use the height of the scar alone [10, 11] or VSS [12] to diagnose HTS. In addition, some papers consider hypertrophic scars alone whereas others combine HTS with contracted or keloid scars to give an overview of pathological scarring [13]. This lack of consistency makes it very difficult to compare studies and draw meaningful conclusions.

Previous literature on the subject of hypertrophic scarring in burns is summarised in Table 4.

**Table 4:** Summary of literature to date

Study	Rate of HTS	Notes/study size
Deitch, 1983 [4]	38% overall	100 patients, adults and children
Spurr and Shakespeare, 1990 [18]	>50% average	Children aged under 5
Zeitlin et al., 1997 [19]	30% overall	91 children, long-term results
Dedovic et al., 1999 [20]	>32% average	779 children, retrospective review of notes
Bombaro et al., 2003 [9]	67% overall	110 major burn survivors
Cubison et al., 2006 [5]	35% overall	337 paediatric scalds
Gangemi et al., 2008 [13]	44% overall	703 adult patients
Chipp et al., current study	17% overall	383 paediatric burns, non-operative treatment

For the purposes of this study, we defined a hypertrophic scar as one which was raised at least 2 mm and had a total mVSS of 5 points or greater. The mVSS was chosen for this study as it is a well-recognised scale and used widely in burn outcome studies [3, 14]. The scale is less suited to large heterogenous scars but we felt it was well suited to this study where the majority of patients had relatively small and well-defined areas of burn scars. It has been criticised in the past for potential operator-dependent errors and interrater variability; we aimed to minimise this in our study by using a small number of experienced burns therapists to assess the scars using the mVSS and each assessment was supplemented with colour photographs for later review if necessary.

Several risk factors have been identified for the formation of HTS. Gangemi et al. showed that female sex, younger age, burn sites on the neck or upper limbs, multiple surgical procedures and meshed skin grafts were all independent risk factors for developing pathological post burn scarring [13]. Hypertrophic burn scars are more common in non-white patient populations [4, 9, 12], and this has also been shown to be true for other types of surgical wounds [15]. Berchialla used these identified risk factors to predict risk of hypertrophic scarring using Bayesian networks [16].

A genetic susceptibility to HTS is suspected but has not yet been proven. Thompson et al. showed that HTS was more common in American Indian/Alaskan Native race (and also in TBSA >20% and facial burns) but were unable to identify the genetic variant responsible for this finding [12]. A recent study from the UK has failed to show a clear link between skin type and incidence of HTS, but this study included small numbers of non-white patients; just 18 of 181 patients had a Fitzpatrick skin type of 4 or above [11].

Our burn centre is located in Birmingham, a city with a very diverse population and an ideal setting for investigating the effect of skin type on hypertrophic scarring. Recent census data shows that approximately half of inhabitants aged 15 years or under (i.e. the population in this study) are of non-white ethnic background and this is predicted to continue rising [17].

This study is of paediatric patients, and it is not clear whether these findings would translate to an adult population, although previous studies would suggest that similar findings could be expected. The relatively high number of patients who did not complete their follow-up may introduce some degree of bias to the study as those patients who fail to attend are likely to be satisfied with the appearance of their scars. However, as far as we are aware, this is the first prospective study of paediatric burns patients on this scale that examines time to healing, skin type and formation of hypertrophic scars.

The rate of HTS in our patient population was relatively low (17.2%) compared to the existing literature. This is likely to be due to the fact that all the patients in this cohort were treated conservatively and would therefore have been predicted to heal relatively quickly. Those patients that are expected to have prolonged healing and therefore a high risk of HTS would usually be managed surgically, and this group will be considered separately.

Our observation is that wounds to certain anatomical sites such as the anterior chest or shoulder region are more likely to form hypertrophic scars but we did not have sufficient numbers in this study to stratify according to both skin type and anatomical location. This would be an interesting point to examine in more detail in future studies. We have no evidence that certain types of dressing or infection led to prolonged healing in any of the groups of patients. Our standard practice is to dress burn wounds with silver-based dressings and to reserve antibiotics for clinically infected wounds. There was no evidence of higher rates of infection in any particular skin type although this factor was not examined as an independent variable for the formation of HTS.

This cohort of patients with relatively small burns, which were mostly treated as outpatients, shows that the incidence of hypertrophic scarring is closely linked to time to healing. We also found a trend towards increased HTS with non-white skin types, particularly skin type 4. Patients with Fitzpatrick skin type 4 have higher rates of HTS overall with rates of almost 13% in all burns healing before 21 days and almost 25% in those which heal between 8 and 14 days; a time period which would traditionally be considered as “safe” with regard to the formation of HTS. We have demonstrated that even in patients who heal before the standard “3-week” period, there is still a considerable risk of developing HTS and this risk appears to be more common in certain skin types although we were unable to demonstrate statistical significance. We believe the morbidity of hypertrophic scarring in children, even with small burns, is significant and should be avoided whenever possible. Traditionally, it was taught that the burn should be healed by 3 weeks in order to avoid unacceptably high rates of hypertrophic scarring. These results show that in certain patients this 3-week target will still lead to a significant risk of HTS with its associated morbidity. The results also show that every additional day to healing leads to a measurable increase in the risk of HTS meaning that every effort should be made to get the patient healed as quickly as possible even if they are approaching or have exceeded the standard 3-week target—the risk of HTS is cumulative with time rather than a linear cut off at 3 weeks. Each additional day to healing gives an odds ratio of 1.138 for developing a hypertrophic scar.

Time to healing appears to be the strongest predictor of HTS according to the data in this study, and this overshadows other risk factors such as skin type and anatomical site. To determine the exact impact of skin type and site of injury, we would need to study a less heterogeneous group of wounds.

## Conclusions

In this prospective study of time to healing and hypertrophic scarring in paediatric burn patients, we have shown that time to healing is strongly associated with the risk of HTS with each additional day to healing conferring an odds ratio of 1.138. We have also shown that a proportion of patients who heal before the traditional 3-week cut off will still develop HTS with its associated morbidity, and this appears to be more common in certain non-white skin types. Although we were not able to prove a statistically significant difference between skin types, we have added to the existing evidence that time to healing is the most important predictor of hypertrophic scarring. We have also demonstrated that the risk of hypertrophic scarring increases on a daily basis from time to healing. These findings are important to all centres treating paediatric burns and especially those with a highly diverse population such as ours.

We believe that the findings of this study emphasise the importance of achieving wound healing as soon as possible in all patients and that we should move away from the traditional “healed by 3 weeks” teaching in order to minimise morbidity for our patients. In conservatively treated paediatric burns, it really does appear that every day counts.

## Abbreviations

HTS: Hypertrophic scar
mVSS: Modified Vancouver Scar Score
POSAS: Patient Observer Scar Assessment Scale
TBSA: Total body surface area
